# Chemometric Analysis of Elemental Fingerprints for GE Authentication of Multiple Geographical Origins

**DOI:** 10.1155/2019/2796502

**Published:** 2019-07-11

**Authors:** Lu Xu, Qiong Shi, Si-Min Yan, Hai-Yan Fu, Shunping Xie, Daowang Lu

**Affiliations:** ^1^College of Material and Chemical Engineering, Tongren University, Tongren 554300, Guizhou, China; ^2^The Modernization Engineering Technology Research Center of Ethnic Minority Medicine of Hubei Province, College of Pharmacy, South-Central University for Nationalities, Wuhan 430074, China; ^3^Shanghai Institute of Quality Inspection and Technical Research, Shanghai 201114, China; ^4^Technology Center, China Tobacco Guizhou Industrial Co., LTD., Guiyang 550009, Guizhou, China

## Abstract

The feasibility of combining elemental fingerprints and chemical pattern recognition methods for authentication of the geographical origins of a Chinese herb, *Gastrodia elata* BI. (GE), was studied in this paper. A total of 210 GE samples were collected from 7 different producing areas. The levels of 15 mineral elements in GE, including Zn, Cd, Co, Cr, Cu, Ca, Mg, Mn, Mo, Ni, Pb, Sr, Fe, Na, and K, were determined using inductively coupled plasma mass spectrometry (ICP-MS). Using the autoscaled data of elemental fingerprints and partial least-squares discriminant analysis (PLSDA), two chemometrics strategies for multiclass classifications, One-Versus-Rest (OVR) and One-Versus-One (OVO), were studied and compared in discrimination of GE geographical origins. As a result, OVR-PLSDA and OVO-PLSDA could achieve the classification accuracy of 0.672 and 0.925, respectively. The results indicate that mineral elemental fingerprints coupled with chemometrics can provide a useful alternative method for simultaneous discrimination of multiple GE geographical origins.

## 1. Introduction

The Chinese herb, “Tianma,” made from the dry tuber of *Gastrodia elata* BI. (GE), has been used as herb in traditional Chinese medicine (TCM) for restoring wind and stopping spasm, calming liver-yang, dispelling wind, and dredging collaterals from ancient times [1, 2]. *Gastrodia elata* is widely distributed in other Asian countries, such as Korea and Japan [3]. Modern scientific research studies have revealed various pharmacological effects of GE, such as anticonvulsant, analgesic, and sedative effects [4, 5]. Clinically, it is mainly used in the treatment of headache, dizziness, numbness of limbs, children's convulsions, epilepsy, tetanus, and so on [6–9]. Herbal chemistry research studies have isolated and identified various physiological active ingredients from GE, including gastrodin, palisarin A, palisarin B, palisarin C, palisarin E, *p*-hydroxybenzyl alcohol, *p*-hydroxybenzaldehyde, polysaccharides, amino acids, and trace elements [10, 11]. Due to the healthy functions and mild actions of GE, it is also used as a popular functional additive in various foods, such as porridges, noodles, dishes, and beverages [12, 13].

Due to the influences of germplasm, cultivation, and environmental and processing conditions, the quality of GE cultivated in different habitats varies greatly, so the quality evaluation of GE is particularly important. Generally, the quality evaluation methods of GE can fall into two classes: capillary electrophoresis [14] and the study of the fingerprint of high-performance liquid chromatography (HPLC) [15, 16]. The index components used for GE quality control include gastrodin, polysaccharides, total flavonoids, amino acids, moisture, and ash [17, 18]. Hazard components in GE, such as sulfur dioxide (sulfur is used by some illegal producers in processing and storage of herbal materials), pesticide residues, and heavy metals, have also been used to inspect and compare the quality of GE samples [19, 20]. HPLC fingerprints also provide a useful tool for quality control of GE by characterizing some of its active components; however, usually the contents of polysaccharides, amino acids, and trace elements were not involved.

As China has a vast territory with diverse geographical, geological, and climate conditions, the types and contents of elements in soils of different regions are different. Moreover, different elements play different roles in herb growth and have different effects on the biosynthesis, accumulation, and distribution of various components in herbs [21, 22]. Therefore, the levels of mineral elements in herbs from different regions are generally not the same, which can provide some useful chemical information to distinguish the geographical origins of herbs [23, 24]. The objective of this work was to develop a practical and effective method by analysis of elemental fingerprints to authenticate the geographical origins of GE samples. The inductively coupled plasma mass spectrometry (ICP-MS) method was used to determine the levels of 15 mineral elements in GE samples. Moreover, two different chemometric strategies for multiclass classifications, One-Versus-Rest (OVR) and One-Versus-One (OVO) [25], were applied and compared to improve the classification performances of mathematical models.

## 2. Materials and Methods

### 2.1. Collection of GE Samples

A total of authentic 210 GE samples from 7 major producing areas were provided by local herb planting bases and Kangqi Medical Plant Development Co., Ltd. (Dejiang, Tongren, China). The 7 producing areas included Hubei (30 samples), Anhui (30 samples), Yunnan (30 samples), Shanxi (30 samples), Guizhou (30 samples), Henan (30 samples), and Gansu (30 samples).

### 2.2. Reagents and Standard Solutions

Standard reserve solutions (1000 *μ*g/mL) of Zn, Cd, Co, Cr, Cu, Ca, Mg, Mn, Mo, Ni, Pb, Sr, Fe, Na, and K were purchased from the National Standard Material Center of China. Secondary distilled water was used in preparing all the sample solutions. For Zn, Cd, Co, Cr, Cu, Mo, Ni, Pb, Sr, and Fe, the serial contents of nitric acid acidified standard solutions were 0. 00, 0.10, 0.50, 1.00, and 5.00 *μ*g/mL; for Ca, Mg, Mn, Na, and K, the serial contents were 0. 00, 1.00, 10.00, 50.00, and 100.00 *μ*g/mL.

### 2.3. Digestion of GE Samples

The GE samples were dried, ground, and digested by a Mars 5 Microwave Digestion System (CEM Corp., USA). About 0.5 g GE powder was precisely weighed and put into a Teflon digestion tank with 7 mL nitric acid (65%, w/w) and 2 mL H_2_O_2_ (30%, w/w). The digestion procedure is summarized in Table 1. In order to reduce blank interferences, all glass instruments and the digestion tank were soaked overnight with nitric acid (10%, w/w), washed repeatedly with deionized water, rinsed with secondary distilled water, and dried naturally.

### 2.4. Determination of Elemental Levels by ICP-MS

Levels of the 15 inorganic elements in GE samples were analyzed using an Agilent 7500a ICP-MS Series inductively coupled plasma mass spectrometer (Agilent Technologies, Santa Clara, CA, USA). The working parameters of the spectrometer was as follows: carrier gas, argon (700 kPa); circulating water, 15–20°C & 250–400 kPa; exhaust volume, 7.0 m^3^/min; absorption rate, 0.3 r/s; absorption time, 30 s; and balance time, 45 s. The elemental contents were determined using the least squares standard curve method.

### 2.5. Chemometrics and Software

Outliers are observation points that are distant from other observations. Obviously, outliers can cause serious problems in statistical analysis. For classification modeling, outliers in the learning set would cause bias in the model parameters or even breakdown of the model, while outliers in the prediction objects would result in false results concerning model performance. Generally, the presence of multiple outliers can have masking effects, that is, some outliers may look like normal data points. In this work, the robust Stahel–Donoho estimate (SDE) of outlyingness [26] was used for outlier diagnosis in GE samples. SDE computes repeated projections of each object onto randomly selected unit vectors. The SDE outlyingness can be derived from the robust location and scatter estimators of projections.

The Kennard–Stone (K-S) algorithm [27] was used to divide the data into a learning set and prediction set. Considering the distribution heterogeneity of GE from different geographical origins, the K-S method was independently applied to each class to split it into learning and prediction objects. For discriminant analysis, learning and prediction objects of different geographical origins were merged into the final learning and prediction sets.

Two different strategies for multiclass classification, One-Versus-One (OVO) and One-Versus-Rest (OVR), were applied to discrimination of GE geographical origins. Both OVR and OVO solve a multiclass problem by using a set of two-class classifiers. For *m* groups to be classified, OVR builds *m* two-class (One-Versus-(*m* − 1)) models. For the *i*th (*i* = 1, 2, 3,…, *m*) model, the group *i* is denoted as +1 and the other (*m* − 1) groups are denoted as −1. A prediction object will obtain a vector of *m* predicted responses from the above *m* models and will be classified into the group corresponding to the largest response value. For OVO, two-class classifiers are built between every pair of the *m* groups, so there are *m*(*m* − 1)/2 OVO classifiers altogether. A test object will be predicted by all the *m*(*m* − 1)/2 models and assigned using a majority voting.

Partial least-squares discriminant analysis (PLSDA) [28, 29] is a classification technique related to partial least squares (PLS). For binary PLSDA, a dummy response vector of +1 (class A) and −1 (class B) is constructed to represent each object in the feature matrix. The cutoff value of predicted response values can be set to be 0, namely, an object with a predicted response value higher/lower than 0 would be assigned to class A/B. An important issue of PLSDA was to estimate the model complexity or the number of significant latent variables (LVs). In this work, Monte Carlo cross validation (MCCV) [30] was used to estimate model complexity by minimizing the error rate of MCCV (ERMCCV) defined as follows:

(1) $$\begin{array}{c} \text{ERMCCV}=\frac{\sum_{i=1}^{K}M_{i}}{N}, \end{array}$$

where *K* is the number of random data splitting by MCCV; *M*_*i*_ is the number of misclassified objects for the *i*th data splitting; and *N* is the total number of test samples in multiple data splitting. Classification accuracy was used to evaluate and compare the performance of OVO-PLSDA and OVR-PLSDA:

(2) $$\begin{array}{c} \text{Accu}=\frac{N_{\text{C}}}{N_{\text{P}}}, \end{array}$$

where *N*_P_ is the size of test set and *N*_C_ is the number of correctly predicted objects.

All the data processing and chemometric analysis were performed on MATLAB 7.0.1 (Mathworks, Sherborn, MA, USA). The K-S algorithm was performed using the codes included in the TOMCAT MATLAB toolbox [31]. All the other algorithms including PLSDA and MCCV were performed using self-compiled MATLAB codes.

## 3. Results and Discussions

### 3.1. Elemental Fingerprints of GE

**Figure 1:**
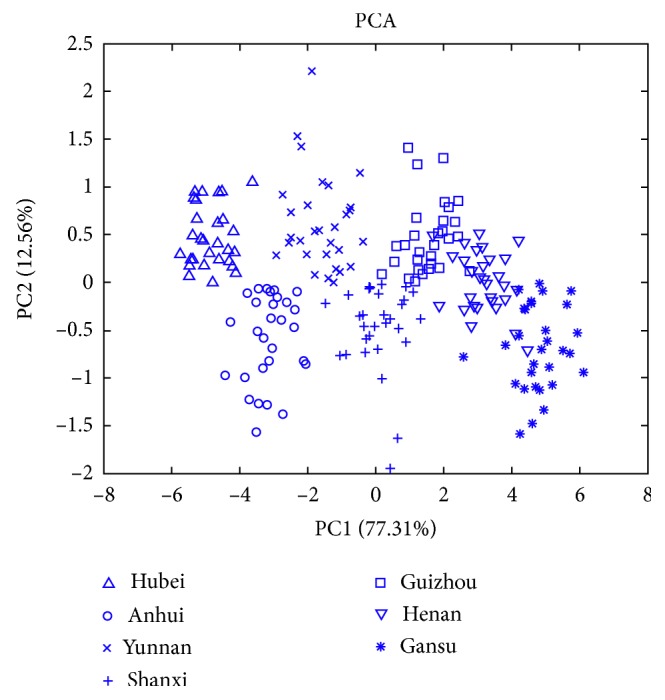
The scores of the first two principal components for GE samples from 7 geographical origins.

**Figure 2:**
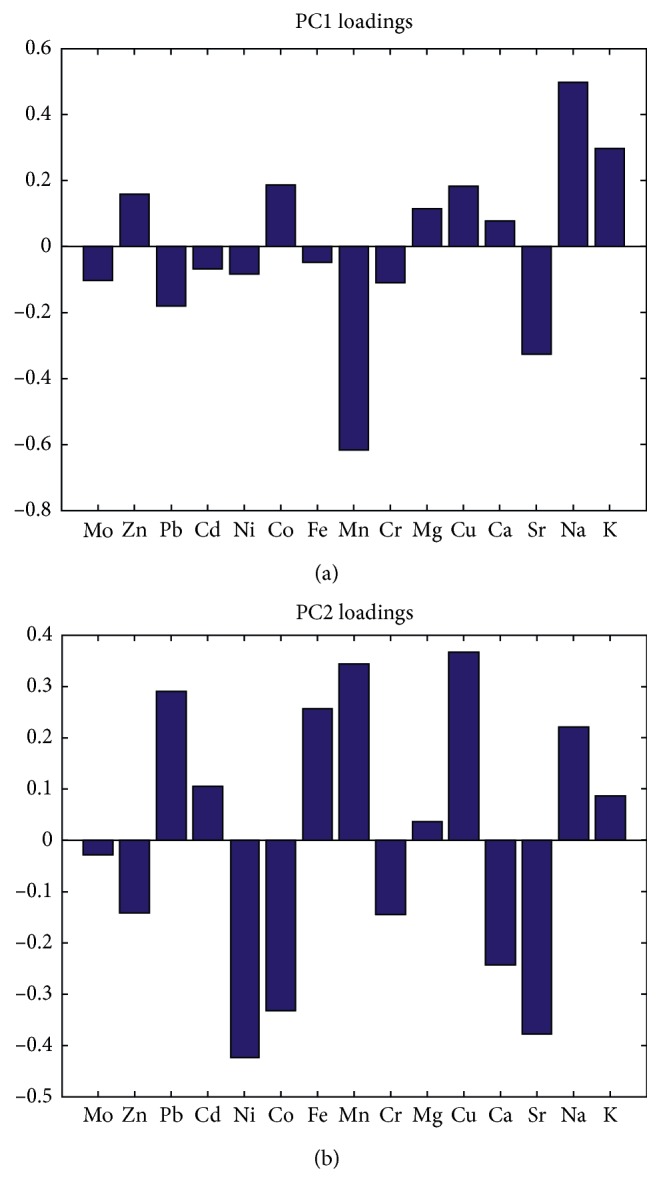
The loadings of PC1 77.31% (a) and PC2 12.56% (b) for GE samples from 7 geographical origins.

The ICP-MS analysis results of the 15 elements for GE samples from different geographical origins are listed in Table 2. Because the data scales of the 15 elements were different, the level of each element was autoscaled (mean-centered and rescaled to have a unit standard deviation) for further analysis. For exploratory analysis, principal component analysis (PCA) was used for dimension reduction of the data (Figure 1). Two principal components were selected to explain more than 89.87% of the total data variances (PC1 77.31%, PC2 12.56%) of the original GE samples ICP-MS from 7 geographical origins. The first 2 PCs can give some separation of the 7 classes although overlapping exists. As is shown in Figure 2, the most positive influence along the PC1 direction can be associated with Na and K, while the most prominent negative impact can be attributed to Mn and Sr. Connecting this with the score plot, a significant increase in Na and K and significant decrease in Sr and Mn can be expected, going from the samples that belong to the Hubei region towards those originating from the Gansu region. Similarly, the highest positive impact along the PC2 direction is observed in the case of Pb, Fe, Mn, Cu, and Na, while the prominent negative impact is observed in the case of Ni, Co, Ca, and Sr. Connecting this with the score plot, one may expect higher content of Pb, Fe, Mn, and Cu in the samples from Hubei, Yunan, and Guizhou regions, while Ni, Co, Ca, and Sr should be dominant in samples originating from Anhui, Shanxi, and Gansu regions. Supervised classification models were required to obtain accurate classification of the 7 classes.

### 3.2. Classification Results

For outlier diagnosis, robust SDE outlyingness was computed for each of the 7 classes. In this work, the number of random projections of SDE was 1000. According to the 3σ rule, SDE outlyingness over 3 is very likely to indicate the presence of an outlier. All the 4 detected outliers were excluded from further data analysis. After removal of outliers, the K-S method was performed on the remaining data of each class to split it into training and test objects, which were combined to form the final learning and prediction sets as shown in Table 3. Finally, a training set of 139 objects and a test set of 67 objects were obtained for training and validation of multiclass PLSDA models.

For both OVO and OVR, MCCV was used to estimate the number of LVs for two-class PLSDA models. In this work, the number of random data splitting by MCCV was 100, and for each data splitting, 30% of data were left out for model validation. The classification results of the 7 classes of GE samples by OVR-PLSDA and OVO-PLSDA are summarized in Table 4. By OVR-PLSDA, the total classification accuracy of the 7 classes was 0.672. The poor prediction results of OVR-PLSDA can be attributed to the following reasons. As shown in Table 4, the average complexity of the 1-VS-6 PLSDA classifiers was 5.29. The high model complexity is usually related to a poor generalization performance. Moreover, because the competitive strategy of OVR involves selecting the largest predicted responses among 7 1-VS-6 classifiers, the final decision-making will be susceptible to class overlapping and submodel errors. Another reason may be the uneven class sizes of the 1-VS-6 models, which can cause bias of the classification cutoff value, although the boundary bias had been corrected [32].

**Figure 3:**
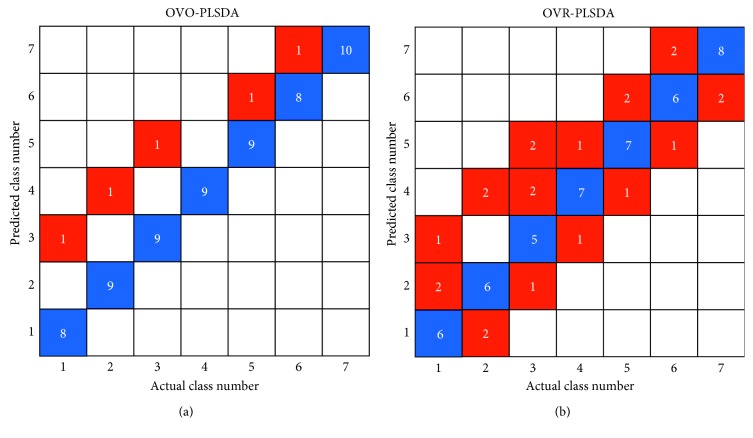
Prediction results for GE samples from 7 geographical origins using elemental fingerprints by OVO-PLSDA (a) and OVR-PLSDA (b) (class labels: 1, Hubei; 2, Anhui; 3, Yunnan; 4, Shanxi; 5, Guizhou; 6, Henan; and 7, Gansu).

**Figure 4:**
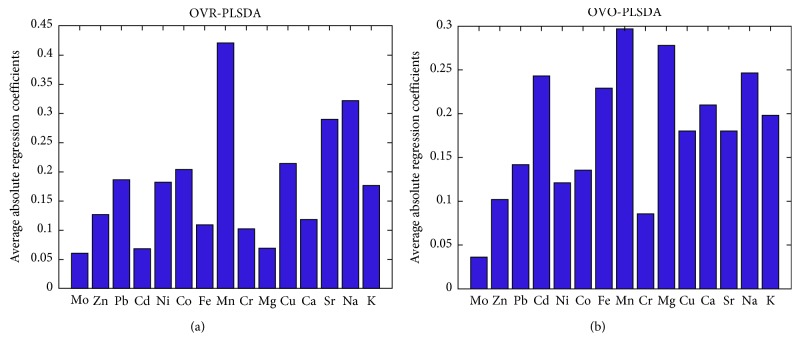
The loading diagrams of the OVR-PLSDA (a) and OVO-PLSDA (b) for GE samples from 7 geographical origins.

The prediction results by OVR-PLSDA and OVO-PLSDA are also shown in Figure 3. For the OVO-PLSDA, the total classification accuracy of the 7 classes was 0.925. The average LVs number of OVO-PLSDA submodels was 2.43. Compared with OVR-PLSDA, the performance of OVO-PLSDA was significantly improved. This can be attributed to the lower model complexity and better classification efficiency of OVO subclassifiers. Figures 4(a) and 4(b) are loading diagrams of the OVR-PLSDA and OVO-PLSDA models, respectively. It can be found that Sr, Na, and Mn have a greater contribution to the geographical origin identification of GE samples in the OVR-PLSDA model, followed by the elements Co and Cu. However, more elements in OVO-PLSDA are considered to be important indicators for the determination of GE samples, such as Cd, Fe, Mn, Mg, Ca, Na, and K. Moreover, for the training data set, because the class sizes of the 7 classes are nearly even (9 or 10 objects in each class), the estimation of classification boundaries of subclassifiers by OVO-PLSDA would be more reliable than by OVR-PLSDA.

## 4. Conclusion

This paper describes the application of ICP-MS elemental fingerprint and chemometrics to the discrimination of the geographical origins of GE samples. The concentration levels of 15 mineral elements (Zn, Cd, Co, Cr, Cu, Ca, Mg, Mn, Mo, Ni, Pb, Sr, Fe, Na, and K) were interpreted using techniques such as PCA, OVR-PLSDA, and OVO-PLSDA. In addition, the OVO-OLSDA model, which was carried out with 7 geographical origins for GE samples, may provide a better prediction ability for samples that are outside of the calibration dataset than the OVR-PLSDA model. This approach improved classification performance with overall accuracy of 0.925, which provided a robust approach for evaluation of the geographical origins of GE samples.

## Figures and Tables

**Table 1:** Selected parameters of microwave digestion system.

Steps	Power (W)	Heating-up time (min)	Temperature retaining (min)	Temperature (°C)
1	1000	15	10	100
2	1000	10	10	150
3	1000	10	10	200
4	1000	5	15	230
5	1000	2	15	240

**Table 2:** Average contents, standard deviations (SD), detection limits, and relative standard deviations (RSD) of the 15 mineral elements in GE samples.

Elements	Average levels (SD) (*μ*g/g)	Detection limit (*μ*g/g)^a^	Average RSD (%) (*n*=6)^b^
Mo	0.51 (0.19)	0.05	1.9
Zn	15.13 (3.18)	0.01	0.8
Pb	0.18 (0.05)	0.01	1.3
Cd	0.08 (0.03)	0.01	2.3
Ni	1.13 (0.36)	0.01	3.6
Co	0.16 (0.05)	0.01	2.1
Fe	52.16 (19.22)	0.6	0.9
Mn	11.68 (4.02)	0.01	1.1
Cr	0.58 (0.22)	0.04	2.6
Mg	183 (59)	0.01	1.0
Cu	1.51(0.36)	0.03	1.9
Ca	411 (119)	0.7	0.7
Sr	11.16 (3.18)	0.01	2.6
Na	116 (38)	0.1	1.1
K	4668 (1066)	2.0	0.8

^a^The levels corresponding to a signal intensity of 3*σ* in the standard curve, where *σ* was computed from 11 repeated measurements of the blank. ^b^Average RSD across samples of different geographical origins.

**Table 3:** Outlier removal and splitting of the data into training and test objects.

Geographical origins	Sample size	Number of outliers	Training objects	Test objects
Hubei	30	1	20	9
Anhui	30	0	20	10
Yunnan	30	0	20	10
Shanxi	30	1	20	9
Guizhou	30	0	20	10
Henan	30	2	19	9
Gansu	30	0	20	10
Sum	**210**	**4**	**139**	**67**

**Table 4:** Classification results of GE samples from 7 geographical origins using elemental fingerprints by OVR-PLSDA and OVO-PLSDA.

Models	ERMCCV	Average LVs	Accuracy
OVR-PLSDA	0.176	5.29	0.672
OVO-PLSD	0.066	2.43	0.925

## Data Availability

The data used to support the findings of this studyare available from the corresponding author upon request by interested readers.
